# Differences in Effort, Reward, Grit, Burnout, and Continuous Exercise Intention: Assessing the Effort-Reward Imbalance among Korean Athletes

**DOI:** 10.5114/jhk/173018

**Published:** 2023-10-27

**Authors:** Inkyoung Park, Wi-Young So, Eui-Jae Lee

**Affiliations:** 1 Department of Sport Science, Seoul National University of Science and Technology, Seoul, Republic of Korea.; 2 Sports Medicine Major, College of Humanities and Arts, Korea National University of Transportation, Chungju-si, Republic of Korea.; 3 Department of Physical Education, Graduate School of Education, Sogang University, Seoul, Republic of Korea.

**Keywords:** mental health, questionnaire, performance, psychology

## Abstract

Although studies have shown that an imbalance between effort and reward in the workplace negatively affects an individual’s physiological and mental health, few have looked at how this imbalance may affect the mental state of athletes. Therefore, this study aimed to evaluate the importance of the effort-reward imbalance (ERI) in athletes by examining whether psychological variables would differ depending on the ERI. To accomplish this, 795 registered collegiate athletes were recruited. Of them, 227 and 230 responses with the ERI in the bottom and in the top 30% of the ERI scale were selected to compare groups with a high and a low ERI. Athletes completed a self-reported 64-item questionnaire (general characteristics: 5; effort: 14; rewards: 14; grit: 12; burnout: 15; and continuous exercise intention: 4 items). Data analysis included reliability and validity using the Jamovi and SPSS/AMOS software. The results showed no significant differences in effort, reward, grit, burnout, and intention to continue to exercise based on gender, weekly training frequency, and hours of training per day. There were differences in effort, reward, grit, burnout, and intention to continue exercising based on the presence of a professional league. Additionally, differences were found in effort, reward, grit, burnout, and intention to continue exercising between the low and high ERI groups. There was a clear difference between the mental state of those who were satisfied with their effort and reward, and those who were not. However, given that the two groups spent approximately the same amount of time exercising each week, the difference may have been more a function of the psychology of athletes than an actual difference in effort and reward. As our findings confirmed that the level of the ERI in athletes is related to their mental state, further research is necessary to identify and control factors that affect the ERI in athletes.

## Introduction

Theory related to behavioral practice, including rational behavior theory, explains that an individual's level of intention to act has an important effect on behavioral practice (Bagozzi, 1992; Rhodes and Yao, 2015). It is also said that a long time difference between intention and practice reduces the influence of that intention (Biddle and Mutrie, 2008); however, the influence of the intention to practice or continue to act on behavior remains important. Therefore, in health research, the intention to sustain is considered an important variable in many areas. However, in Korea, research on the intention to continue sports is sparse. To date, most studies on Korean athletes have focused on factors that affect performance rather than the intention to continue exercising (Kim, 2015; Lee and Lee, 2023; Yoon, 2022). However, recently, the importance of encouraging young athletes to continue exercising has been recognized, as only few young athletes are discovered and nurtured into professional players, which is a lengthy process (Chamera et al., 2023; Kong, 2022; Oh and Yang, 2017; Pires and Ugrinowitsch, 2021). Athletes are more likely to stop exercising during their participation if they do not find joy in the sport itself or if their motivation to participate decreases (Gonzalez-Hernandez et al., 2023; Weiss and Amorose, 2008). This will also affect their performance, result in potential withdrawal, and exacerbate exhaustion (Park et al., 2017). Therefore, for young athletes, the intention to continue exercising is as important as their performance. Thus, it is crucial to identify ways to encourage them and increase their intention to continue exercising (Diaz-Garcia et al., 2021).

The effort-reward imbalance (ERI) model described by Siegrist (1996) suggests that the results obtained in relation to what one is doing are evaluated by a reciprocal relationship between effort and reward. In other words, when you feel that the reward is less than the effort you make, an imbalance in reciprocity occurs, which causes stress related to your actions. Stress generated in this manner negatively affects physical and mental health. The ERI model, which has been mainly applied in measuring job stress, has recently been suggested for college student school life (Wege et al., 2017), jockeys (Kathleen et al., 2017), and athletes (Hong et al., 2023). The ERI model has been reported to be useful for explaining many social phenomena, although not all situations. Siegrist (1996) explained that in the current ERI model, when there is no alternative or when there is a strategy for future gains and high motivation, such as immersion or fun, the situation continues to work. Moreover, several scholars state that the quality of time and effort required to learn a skill are both important to become an expert in the field (Kuhlmann and Ardichvili, 2015; Persky and Robinson, 2017). For athletes, this means investing time and effort to reach the highest performance level. However, it is not easy to get a satisfactory reward because the top positions are limited. As such, athletes may be aware of this ERI; however, it is unclear whether this results in psychological issues.

Previous studies have reported that a prolonged imbalance between effort and reward affects absenteeism and subsequent mental symptoms such as stress, depression, burnout, and job dissatisfaction, as well as physical symptoms, such as cardiovascular and musculoskeletal disorders (Devreux et al., 2012; Rugulies et al., 2017; Willis et al., 2008). Considering athletes, significant effort is expended over an extended period. Therefore, they may be exposed to an imbalance between effort and reward over a long time as well. However, there is limited empirical research on whether the ERI model is applicable to athletes.

Therefore, this study aimed to identify the existing differences between the ERI, weekly training time and various mental variables of athletes depending on individual characteristics to confirm whether the ERI is important to athletes. We also intended to confirm the difference in the athletes' effort, reward, grit, exhaustion, intention to continue exercise and weekly training time between the high and low ERI groups. The research results obtained in this way can also determine whether the effort-reward imbalance model is applicable to athletes. Furthermore, our results may provide the basis to identify ways to alleviate the ERI perceived by athletes.

## Methods

### Participants

For our study, we recruited a total of 795 college student athletes from universities in Seoul, Gyeonggi-do, Gangwon-do, Chungcheong-do, and Jeolla-do, Korea registered by 2022. To identify the sample size, we used analysis of variance, an anticipated statistical power of 0.90 and α-error probability of 0.05 with an effect size of 0.30. A minimum sample size of 382 was identified (G*Power 3.1.9.7, Heinrich-Heine-University, Düsseldorf, Germany). However, because we intended to compare groups in top 30% and bottom 30% of the ERI scale, we selected a sample size of 800, considering the probability of omission; thus, twice the 382 derived from the G*Power results. Five individuals were excluded from our analysis owing to insufficient or inconsistent responses. We used convenience sampling to solicit athletes and a self-reported questionnaire for the evaluation.

The frequency was confirmed by measuring the effort-reward ratio of the study participants from the collected data. We then established two groups, one with results in bottom 30% of the ERI scale and he second one with the results in top 30% of the ERI scale. There were 227 athletes in the low ERI group (men: 198, women: 29), with an average age of 20.23 ± 1.18 years. The frequency of weekly exercise in that group was 5.67 ± 0.71 days, the average daily training time was 4.25 ± 1.56 hours, and the weekly exercise time was 24.27 ± 10.05 hours. In the high ERI group, there were 230 athletes (men: 208, women: 22) with an average age of 20.10 ± 1.22 years. The frequency of weekly exercise in this group was 5.87 ± 0.69 days, the average daily training time was 4.10 ± 1.47 hours, and the weekly exercise time was 24.32 ± 9.66 hours. Only responses selected from the top and bottom 30% groups of the ERI were used for ANOVA, and those not within this range were not considered. Moreover, athletes practiced 23 different sports disciplines (professional leagues: basketball, soccer, volleyball, baseball; non-professional leagues: rugby, wrestling, badminton, boxing, ice skating, softball, swimming, skiing, Korean wrestling, archery, weightlifting, judo, cycling, rowing, gymnastics, taekwondo, tennis, and handball).

### Instruments

The applied questionnaire consisted of 64 questions: five on general characteristics, 14 on effort, 14 on reward, 12 on grit, four on exercise continuous intention, and 15 on exhaustion. However, based on item, reliability, and construct validity analyses, we looked at all 14 effort items and all 4 exercise continuous intention items, but only 13 items related to reward, 9 to grit, and 9 items of exhaustion. The reliability and validity analyses are shown in Table 1.

**Table 1:** Content, validity, and reliability of the survey.

Items				Loading	Reliability	Suitability	
Effort	A		I train individually (besides team training). I do extra-training sessions before and after scheduled team training. I try more than other athletes. I do additional strength training and conditioning on my own. I spend more time on training than other athletes do.	0.775 0.834 0.600 0.685 0.713	0.857	0.878	*χ*^2^ = 208.444 df = 71 *p* < 0.001 SRMR = 0.049 CFI = 0.954 TLI = 0.941 RMSEA = 0.065
	B		I am always polite with all colleagues and teammates. I try to keep a good relationship with my teammates. I try to understand the difficulties my teammates are facing.	0.770 0.860 0.834	0.784		
	C		I take some supplements (nutritional supplements). I try to eat healthy to take care of my body. I care about my nutritional management.	0.616 0.964 0.457	0.761		
	D		By watching video footage, I analyze my individual performance and my competition. I analyze video clips of outstanding athletes. I do imagery training (visualization training).	0.869 0.818 0.555	0.817		
Reward	E		As an athlete, my future is promising. If I continue to perform as now, I will be recognized in the field. If I continue to perform, my contract period will be guaranteed. If I continue to perform, I will be able to continue my career at the university or professional level. If I continue to perform, I can have a socially stable life. If I continue on this trajectory, I will be able to reach my goals.	0.736 0.832 0.873 0.915 0.815 0.830	0.938	0.927	*χ*^2^ = 158.589 df = 59 *p* < 0.001 SRMR = 0.037 CFI = 0.978 TLI = 0.971 RMSEA = 0.061
	F		I have received praise and recognition from other athletes and coaches. I believe that I am being treated fairly based on my ability/performance. During training, I get attention and instruction from the coach. I am fairly treated on the team.	0.654 0.715 0.784 0.751	0.850		
	G		I think it is a reward for my efforts when I get an individual award. I believe that improvement in my technical skills is a reward for my hard work. When I play without regret there are rewards for my hard work.	0.647 0.936 0.696	0.836		
Grit	H	I often set a goal but later choose to pursue a different one (a). I become interested in new pursuits every few months (a). My interests change from year to year (a). I have been obsessed with a certain idea or a project for a short time but later lost interest (a), (b). New ideas and projects sometimes distract me from previous ones (a), (b). I have difficulty maintaining my focus on projects that take more than a few months to complete (a), (b).		0.637 0.616 0.649	0.688	0.785	*χ*^2^ = 52.694 df = 26 *p* < 0.001 SRMR = 0.029 CFI = 0.984 TLI = 0.978 RMSEA = 0.047
	I	I have achieved a goal that took years of work. Setbacks don’t discourage me. I am a hard worker. I finish whatever I begin. I have overcome setbacks to conquer an important challenge. I am diligent.		0.792 0.737 0.746 0.779 0.718 0.773	0.892		
Burnout	J	I’ve accomplished many worthwhile things in sports (a). I have not achieved much in sports. It seems that no matter what I do, I don’t perform as well as I should. I am not performing up to my ability in sports (b). I feel successful in sports (a), (b).		0.722 0.378 0.380	0.688	0.885	*χ*^2^ = 87.85 df = 23 *p* < 0.001 SRMR = 0.047 CFI = 0.965, TLI = 0.946, RMSEA = 0.079
	K	I feel so tired from my training that I have trouble finding energy to do other things. I feel overly tired from my sport participation. I feel “wiped out” from sports. I feel physically worn out from sport (b). I am exhausted by the mental and physical demands of sport (b).		0.769 0.823 0.744	0.828		
	L	I don’t care as much about my sports performance as I used to. I’m not into sports like I used to be. I feel less concerned about being successful in sports than I used to. The effort I invest in sports would be better invested in other activities (b). I have negative feelings toward sports (b).		0.779 0.924 0.775	0.861		
Exercise continuous intention		I will continue to do the sports I do. I want to continue the sports I do in any case. Surely, I have the will to continue this sport next month. I want to increase my sport time.		0.934 0.887 0.828 0.519	0.859	*χ*^2^ = 6.87, df = 2 *p* = 0.030 SRMR = 0.010 CFI = 0.990 TLI = 0.990 RMSEA = 0.070	

A: training reinforcement efforts, B: interpersonal relationship efforts, C: nutrition management efforts, D: cognitive and psychological efforts, E: future stability, F: social support, G: positive growth, SRMR: standardized root mean square residual, CFI: comparative fit index, TLI: Turker-Lewis index, RMSEA: root mean square error of approximation assessed through confirmatory factor analysis H: Consistency of Interests, I: Perseverance of Effort J: Reduced sense of accomplishment, K: Physical/emotional exhaustion, L: Devaluation of sports practice. SRMR: standardized root mean square residual, CFI: comparative fit index, TLI: Turker-Lewis index, RMSEA: root mean square error of approximation (a) Items were reverse scored; (b) Items were excluded from the analysis assessed through confirmatory factor analysis

#### General Characteristics

We asked basic questions covering age, gender, the type of sport, frequency of weekly exercise, and daily training time of athletes. Athletes self-reported all the answers in the questionnaire. Among participants, 291 players were in professional leagues while 504 players were not. For the frequency of weekly training, 254 athletes trained two to five days, 443 athletes trained six days, and 98 athletes trained seven days; 304 athletes trained one to three hours, 322 athletes trained four to five hours, and 169 athletes trained for six hours or more. By multiplying the frequency of weekly training by the daily training time, we confirmed that the average training time was 24 hours per week.

#### Effort-Reward Imbalance

We used the Korean effort and reward scale developed by Park and Kim (2021) to measure the degree of the ERI in athletes. We measured effort and reward as perceived by athletes based on 14 questions on effort under four factors (training reinforcement, interpersonal relationship, nutrition management, and cognitive and psychological efforts) and 13 questions on reward under three factors (future stability, social support, and positive growth). Responses to these questions were scored on a five-point Likert scale (1 = strongly disagree to 5 = strongly agree). As a result of verifying the construction validity, we found that the model suitability of questions for effort (4 factors, 14 questions) and reward (3 factors, 13 questions) was appropriate (effort: *χ*^2^ = 208.44; df = 71; *p* < 0.001; Q = 2.936; standardized root mean square residual [SRMR] = 0.049; Turker-Lewis index [TLI] = 0.941; comparative fit index [CFI] = 0.954; root mean square error of approximation [RMSEA] = 0.065; reward: *χ*^2^ = 158.59; df = 59; *p* < 0.001; Q = 2.688; SRMR = 0.037; CFI = 0.978; TLI = 0.971; RMSEA = 0.061). Following the methods of Siegrist (2012) and Wege et al. (2017), we determined the individual degree of the ERI, and the effort-reward ratio, by dividing the total score for effort by the total score for reward. The suitability of this method was also suggested by Siegrist et al. (2009).

#### Grit

We used a scale that verified validity by translating the grit questionnaire developed by Duckworth et al. (2007), consisting of 12 questions and 2 factors: maintaining interest and continuing efforts. In the scale, questions under the “maintenance of interest” factor were analyzed through reverse scoring, where the higher the score, the higher the level of grit. Responses were scored on a five-point Likert scale (1 = strongly disagree to 5 = strongly agree). As a result of verifying the grit questions through confirmatory factor analysis, we used the two factors and nine questions (*χ*^2^ = 52.694; df = 26; *p* = 0.001; Q = 2.027; SRMR = 0.029; CFI = 0.984; TLI = 0.978; RMSEA = 0.047).

#### Continuous Exercise Intention

To evaluate athletes’ intentions to continue exercising, adapted from the continuous intention questionnaires used by Ajzen (2006) and Hoffman and Novak (1996), we followed the questions used by Park et al. (2021). Those comprised four questions on continuing to exercise, with a higher score indicating a higher intention to continue exercising. A five-point Likert scale (1 = not like me at all to 5 = very much like me) was used to measure the responses. By verifying the construct validity of the continuous intention question, model suitability was found to be appropriate (*χ*^2^ = 6.873; df = 2; *p* = 0.032; Q = 3.436; RMR = 0.017; CFI = 0.996; TLI = 0.987; RMSEA = 0.073).

#### Burnout

To confirm burnout in athletes in Korea, previously, Han and Kim (2011) translated the Athlete Burnout Questionnaire developed by Raedeke and Smith (2001) and used a scale with high suitability after validation. The scale is intended to assess athletes’ exhaustion and consists of three factors (physical and emotional exhaustion, a reduced sense of achievement, and devaluation of sports practice). Questions 1 and 14 (deleted in the analysis process) were scored as reverse questions and used for analysis under the “reduced sense of achievement” factor. Responses were scored on a six-point Likert scale (1 = very strongly disagree to 6 = very strongly agree). After verifying the composition validity of the burnout question, three factors and nine questions were used (*χ*^2^ = 87.853; df = 23; *p* < 0.001; Q = 3.820; SRMR = 0.047; CFI = 0.965; TLI = 0.946; RMSEA = 0.079).

#### Procedures

Athletes who expressed their willingness to participate in the study signed a consent form which indicated the purpose of the study and the contents of the survey. The form also stated that the survey could be stopped at any time. For those willing to participate, a small gift was provided. The collected surveys were checked and converted into data suitable for analyses, excluding unusable data and those who did not respond truthfully to the survey (e.g. excluding unusable data and the data that showed the same response to most surveys). The study was conducted in accordance with the guidelines of the Declaration of Helsinki and the protocol was approved by the Institutional Review Board of the Seoul National University of Science and Technology (IRB approval number: 2021-0005-01; approval date: 3 June 2021).

### Statistical Analyses

The data were analyzed using Jamovi 1.6.15 and SPSS 23.0/AMOS 23.0 (IBM Corp., Armonk, NY, USA). First, suitability was confirmed through item reliability and exploratory and confirmatory factor analyses of the measurement tool. Second, to confirm differences according to the ERI, the lower and upper 30% groups were established. Responses not included in both groups were not used for the independent *t*-test. An independent sample *t*-test was conducted to confirm differences in psychological variables between the two groups. All statistical significance levels were set at *p* < 0.05.

## Results

### Difference by Variables

Differences in effort, reward, ERI, grit, exhaustion, and intention to continue exercising according to gender, professional league sports, weekly training frequency, and weekly training time are presented in Table 2. Significant difference in psychological variables according to gender was only reflected in the sub-variables under effort: training reinforcement (*t* = 13.379; *p* < 0.001) and overall effort (*t* = 6.208; *p* = 0.013). Variables such as “training reinforcement” showed higher scores for male than for female athletes, but in reality, female athletes had more weekly training time than male athletes (*t* = 7.086; *p* = 0.008). There were no gender differences in terms of the other variables.

**Table 2:** Differences in psychological variables according to characteristics.

		n	Effort					Reward				ERI
			A	B	C	D	Total	E	F	G	Total	
Gender	Men	709	3.52	4.19	3.48	3.92	3.74	3.13	3.39	4.01	3.46	1.10
			0.73	0.65	0.90	0.78	0.58	0.88	0.70	0.68	0.65	0.19
	Women	86	3.22	4.06	3.48	3.79	3.58	3.08	3.37	3.98	3.42	1.07
			0.60	0.63	0.77	0.69	0.47	0.84	0.73	0.59	0.65	0.19
	t		13.379 ***	2.848	0.001	2.071	6.208 *	0.260	0.101	0.197	0.272	2.896
Professional league sports	Yes	291	3.69	4.26	3.66	4.02	3.88	3.28	3.48	4.06	3.56	1.10
			0.68	0.62	0.86	0.73	0.56	0.84	0.69	0.64	0.62	0.17
	No	504	3.36	4.12	3.39	3.84	3.63	3.03	3.34	3.98	3.39	1.09
			0.72	0.66	0.89	0.79	0.56	0.88	0.70	0.69	0.66	0.19
	t		39.358 ***	8.638 **	17.663 ***	10.517 **	35.415 ***	15.315 ***	6.541 *	2.917	12.560 ***	1.149
Training frequency	2~5 (a)	254	3.35	4.12	3.31	3.86	3.62	3.10	3.42	3.92	3.43	1.07
			0.67	0.58	0.86	0.76	0.54	0.90	0.69	0.67	0.67	0.18
	6 (b)	443	3.52	4.17	3.57	3.94	3.76	3.13	3.40	4.06	3.47	1.09
			0.72	0.68	0.87	0.78	0.57	0.86	0.70	0.67	0.65	0.18
	7 (c)	98	3.66	4.35	3.56	3.90	3.84	3.15	3.28	4.01	3.43	1.13
			0.83	0.64	0.95	0.77	0.60	0.91	0.71	0.64	0.64	0.17
	F		7.487 **	4.434 *	7.773 ***	0.745	7.346 **	0.158	1.576	3.538	0.477	3.668 *
	Scheffe		a<b,c	a,b<c	a<b,c	-	a<b,c	-	-	-	-	a<c
Training time	1~3 (d)	304	3.44	4.12	3.43	3.88	3.67	3.08	3.37	3.98	3.42	1.09
			0.65	0.62	0.85	0.75	0.54	0.85	0.64	0.66	0.62	0.17
	4~5 (e)	322	3.44	4.18	3.46	3.88	3.70	3.03	3.31	3.97	3.38	1.11
			0.78	0.68	0.92	0.81	0.60	0.87	0.70	0.67	0.64	0.18
	6~7 (f)	169	3.65	4.26	3.64	4.01	3.85	3.38	3.59	4.15	3.66	1.07
			0.74	0.64	0.87	0.75	0.55	0.90	0.77	0.68	0.69	0.19
	F		5.309 **	2.784	3.472 *	1.879	5.922 **	9.439 ***	9.546 ***	4.563 *	11.229 ***	2.285
	Scheffe		d,e<f	-	d,e<f	-	d,e<f	d,e<f	d,e<f	d,e<f	d,e<f	-

A: training reinforcement efforts, B: interpersonal relationship efforts, C: nutrition management efforts, D: cognitive and psychological efforts, E: future stability, F: social support, G: positive growth, ERI: effort-reward imbalance* *p* < 0.05, ** *p* < 0.01, *** *p* < 0.001, assessed through one-way analysis of variance

**Table 2:** (cont.) Differences in psychological variables according to characteristics.

		n	Grit			Burnout				Intention	Weekly training time
			H	I	total	J	K	L	Total		
Gender	Men	709	3.31	3.75	3.58	3.03	3.44	2.80	3.09	3.93	24.14
			0.71	0.68	0.53	0.95	0.97	1.11	0.84	0.90	10.03
	Women	86	3.36	3.65	3.53	3.01	3.29	2.86	3.06	3.78	27.22
			0.71	0.71	0.52	0.95	1.05	1.11	0.92	0.90	10.97
	t		0.307	1.881	0.567	0.290	1.913	0.262	0.148	2.027	7.086 **
Professional league sports	Yes	291	3.34	3.82	3.63	2.94	3.32	2.71	2.99	4.01	24.24
			0.72	0.63	0.53	0.92	0.97	1.08	0.82	0.87	11.32
	No	504	3.30	3.69	3.54	3.08	3.49	2.86	3.14	3.85	24.61
			0.71	0.70	0.52	0.97	0.97	1.13	0.86	0.92	9.46
	t		0.474	6.387 *	5.355 *	3.798	5.401 *	3.213	5.832 *	5.205 *	0.239
Training frequency	2~5 (a)	254	3.25	3.67	3.50	3.04	3.39	2.89	3.11	3.77	18.44
			0.72	0.67	0.52	0.97	0.94	1.07	0.85	0.95	7.17
	6 (b)	443	3.37	3.75	3.60	3.04	3.45	2.76	3.09	3.98	26.15
			0.71	0.67	0.54	0.97	1.00	1.14	0.87	0.88	9.35
	7 (c)	98	3.29	3.85	3.62	2.95	3.41	2.78	3.05	3.96	32.50
			0.68	0.72	0.48	0.86	0.97	1.09	0.78	0.85	11.90
	F		2.367	2.580	3.209 *	0.385	0.286	1.167	0.196	4.430 *	101.658 ***
	Scheffe		-	-	a<b,c	-	-	-	-	a<b	a<b<c
Training time	1~3 (d)	304	3.27	3.69	3.52	3.05	3.36	2.85	3.09	3.88	14.75
			0.70	0.63	0.50	0.93	0.90	1.09	0.82	0.89	3.08
	4~5 (e)	322	3.38	3.74	3.59	3.05	3.47	2.83	3.12	3.86	26.07
			0.70	0.70	0.54	0.91	0.99	1.13	0.85	0.92	4.52
	6~7 (f)	169	3.29	3.84	3.62	2.95	3.44	2.67	3.03	4.06	38.92
			0.75	0.69	0.54	1.06	1.07	1.12	0.91	0.87	6.89
	F		2.114	2.934	2.492	0.673	1.014	1.563	0.671	3.163 *	1475.634 ***
	Scheffe		-	-	-	-	-	-	-	d,e<f	a<b<c

H: consistency of interests, I: perseverance of effort, J: reduced sense of accomplishment, K: physical/emotional exhaustion, L: devaluation of sports practice* *p* < 0.05, ** *p* < 0.01, *** *p* < 0.001, assessed through one-way analysis of variance

There were significant differences between groups in various psychological variables between players in sports with professional leagues (basketball, soccer, volleyball, baseball) and those in sports without professional leagues (effort: *t* = 39.358, *p* < 0.001; reward: *t* = 12.560, *p* < 0.001; grit: *t* = 5.355, *p* = 0.001; burnout: *t* = 5.832, *p* = 0.016; and exercise continuous intention: *t* = 5.205, *p* = 0.023). However, there was no difference in weekly training time between athletes competing in professional leagues and their counterparts (rugby, wrestling, badminton, boxing, ice skating, softball, swimming, skiing, Korean wrestling, archery, weightlifting, judo, cycling, rowing, gymnastics, taekwondo, tennis, and handball) (*t* = 0.239, *p* = 0.625).

Regarding differences in psychological variables according to the frequency of weekly training, those with higher training frequency had significantly higher effort factors than those with lower training frequency (*F* = 7.346, *p* = 0.010).

In addition, those with higher training frequency felt a greater ERI than those with lower frequency in weekly training (*F* = 3.668, *p* = 0.026). In addition, grit and exercise continuity intention were statistically higher in the group with high training frequency compared to the one with low training frequency (grit: *F* = 3.209, *p* = 0.041; exercise continuity intention: *F* = 4.430, *p* = 0.012). Depending on the daily training time, those with more training per day showed higher effort, reward factors, and intention to continue exercising than those with less training time per day (effort: *F* = 5.309, *p* = 0.005; reward: *F* = 11.229, *p* < 0.001; continuous exercise intention: *F* = 3.163, *p* = 0.043).

### Differences in Psychological Variables according to the ERI

In our study, we investigated how psychological variables were perceived by the high and low ERI groups (Table 3). As a result, it was found that the group with a high ERI reached significantly higher values in effort factors than the group with a low ERI (*t* = 36.526, *p* < 0.001) and the group with a low ERI received more rewards than the group with a high ERI (*t* = 364.307, *p* < 0.001). Moreover, among the sub-factors of grit, factors of continuing effort (*t* = 6.865, *p* = 0.009) and intention to continue exercising (*t* = 24.851, *p* < 0.001) presented higher values in the lower ERI group than in the higher ERI group. However, exhaustion was higher in the higher ERI group (*t* = 36.864, *p* < 0.001).

**Table 3:** Differences in psychological variables according to the effort-reward imbalance level.

	Group	n	Effort					Reward			
			A	B	C	D	Total	E	F	G	Total
Level of effort-reward imbalance	Low	227	3.32	4.04	3.24	3.78	3.56	3.74	3.85	4.27	3.92
			0.67	0.66	0.90	0.84	0.59	0.79	0.68	0.64	0.62
	High	230	3.58	4.37	3.78	3.99	3.88	2.43	2.92	3.73	2.94
			0.79	0.61	0.82	0.76	0.55	0.69	0.59	0.67	0.47
	t		13.832 ***	29.106 ***	44.497 ***	8.161 **	36.526 ***	360.031 ***	243.298 ***	79.178 ***	364.307 ***

A: training reinforcement efforts; B: interpersonal relationship efforts; C: nutrition management efforts; D: cognitive and psychological efforts; E: future stability; F: social support; G: positive growth** *p* < 0.01, *** *p* < 0.001, assessed through an independent samples *t*-test

**Table 3:** (cont.) Differences in psychological variables according to the effort-reward imbalance level.

	Group	n	Grit			Burnout				Intention	Weekly training time
			H	I	Total	J	K	L	Total		
Level of effort-reward imbalance	Low	227	3.36	3.78	3.62	2.70	3.36	2.44	2.85	4.12	24.27
			0.74	0.69	0.52	0.99	1.06	0.99	0.85	0.82	10.05
	High	230	3.38	3.62	3.52	3.34	3.49	3.13	3.32	3.69	24.32
			0.70	0.68	0.54	0.86	0.94	1.13	0.81	1.01	9.66
	t		0.056	6.865 **	3.631	53.551 ***	1.960	48.124 ***	36.864 ***	24.851 ***	0.165

H: consistency of interests, I: perseverance of effort, J: reduced sense of achievement; K: physical/emotional exhaustion; L: devaluation of sports practice** *p* < 0.01, *** *p* < 0.001, assessed through an independent samples *t*-test

## Discussion

The relatively recently organized ERI model for job stress research (Siegrist, 2001) explains that the principle of reciprocity is important in the role that humans play in social life in the work environment. Among various social lives, reciprocity in the job field is understood as the effort that individuals expend on job activities with the expectation of an adequate reward. If an imbalance between individual efforts and reward recognition persists, negative emotional reactions occur, eventually affecting health (Bosma et al., 1998). This captures the usefulness of the ERI model in various job environments. However, its usefulness for athletes has not yet been confirmed. Therefore, our study evaluated whether the ERI model affects the mental state of athletes and ultimately, their intention to continue to exercise. Based on our analysis, we found that male athletes stated that they expended more effort in strengthening their training than female athletes. Kim et al. (2020) indicate that male athletes are better at self-management related to training than female athletes. However, Heo’s (2003) study shows no significant gender differences. In our study, the size of the classified groups varied by gender, which is worth considering when discussing gender-based outcomes. However, one interesting finding was that even though male athletes mentioned that they put extensive effort into training, female athletes actually spent more time training than male athletes did during the week.

According to the results of professional league sports players and non-professional players, there was no difference in exercise time between the two, although professional league athletes recorded higher effort factors. Female athletes who trained for more hours did not rate their efforts as high, neither did athletes in sports who trained for a similar number of hours and were not part of professional leagues. These results highlight the need for a follow-up study on why participants evaluated their efforts so low despite considerable time spent on practice. In Korea, sports without professional leagues are considered unpopular with the public or unfamiliar to the public. Moreover, in general, the annual salary and compensation systems are poor compared with those of professional leagues. In fact, annual salaries vary greatly, not only in Korea but also globally, depending on the sports and the league. For example, the average annual salary of an NBA player is more than $8 million, while in the East Coast Hockey League, a rookie earns less than $24,000 (Gough, 2023). Hong and Fraser (2021) explain that athletes are also stressed by noncompetitive factors, such as job insecurity, along with stress linked to competition. Athletes who play in environments that emphasize sportsmanship, such as amateurism and fair play, may be unable to achieve financial reward. In fact, rewards, such as salary and future stability, are important for professional athletes. In addition, future stability of the sport plays a significant role for college athletes who have not yet entered the professional status.

Our results showed that players in professional leagues had higher grit as well as a greater intention of continuing to exercise and lower burnout than those who were not in these leagues. This result is partially supported by Hong et al.’s study (2023). Our results also showed that mental variables may vary greatly depending on the sport. Although a slightly different issue, recently, there have been opinions that question the inequality of the compensation system according to gender in sports (Cavil and Jenkins, 2023). Moreover, we should also consider how reward systems differ depending on the popularity of the sport. In our study, those with high weekly training frequency or daily training time recognized higher effort, reward, grit, and exercise continuity intention than those with less training frequency; and the group with a high training frequency also recognized a large ERI. There are only few similar previous studies, however, Granz et al. (2019) reported that higher training was linked to burnout and training intention. Training intensity has also been linked to a correlation with emotional/physical exhaustion (Vetter and Symonds, 2010). Nevertheless, in our study, burnout did not show significant differences between the groups.

In the analysis of differences according to the degree of the ERI, the group with a low ERI recounted a higher level of effort and reward than the group with a high ERI. In addition, we found that athletes with a low ERI responded highly to the grit sub-factors, “perseverance of effort”, and the intention to continue exercising. In contrast, the group with a high ERI showed higher burnout. In short, athletes who were highly aware of the ERI scored low on positive mental factors related to exercise behavior and high on negative mental factors. Although not the same, it was reported that high ERI recognition on the job results in low scores on variables such as job satisfaction and well-being risk (Cho et al., 2021), suggesting that low ERI recognition is related to positive psychology in the job environment. However, a study on Chinese university students reports that a high ERI is closely related to stress (Kequn et al., 2023), while studies on female nurses (Rastjoo and Zandvanian, 2021) and information technology experts (Raju et al., 2022) report high correlations. In our study, the high ERI group had a greater level of burnout than the low ERI group, which is consistent with previous studies. The fact that the athletes’ ERI relates to positive and negative variables which affect their sports situation confirms the value of the application of the ERI model in the sports field, similarly to that in other job areas. However, unlike other jobs, the sports sector requires considerable time and effort to reach a top position and differs from other jobs as the participant may experience a long-term ERI. Therefore, follow-up studies are warranted to evaluate what variables endure during the ERI over the long term.

## Conclusions

Our study measured college athletes’ effort, reward, ERI, grit, burnout, and intention to continue exercising and identified differences in mental variables in groups with a high and a low ERI. We contribute to literature in two meaningful aspects. First, we confirmed that the ERI model can be applied to athletes, similarly to its application in various occupational areas. Therefore, continuous research should be conducted to identify ways to lower the ERI for continuous exercise performance and the mental health of athletes. Second, we confirm that the ERI model is applicable to athletes, but further longitudinal research is required to examine the extent of the ERI for athletes. Moreover, we found a relationship between the effort perceived by athletes and actual exercise time. Female athletes indicated greater actual exercise time, while male athletes indicated less exercise time but evaluated their efforts higher than their female counterparts. Of course, the quality of effort cannot be evaluated only by time factors, but this is an interesting aspect and should be explored in future research. Although the results of this study are interesting, the number of male and female athletes was significantly different; thus, the results should be interpreted with caution. In addition, we believe the results would be more interesting if we evaluated the stress presented in previous studies of the ERI model and the perceived performance presented in the present study in athletes. Therefore, follow-up studies that consider these aspects are necessary.
